# Gestational Breast Cancer – a Review of Outcomes, Pathophysiology, and Model Systems

**DOI:** 10.1007/s10911-023-09546-w

**Published:** 2023-07-14

**Authors:** Mackenzie K. Callaway, Camila O. dos Santos

**Affiliations:** Cold Spring Harbor Laboratory, Cancer Center, Cold Spring Harbor, NY USA

**Keywords:** Pregnancy, Breast Cancer, Therapy, Stroma, Extracellular Matrix, Models

## Abstract

The onset of pregnancy marks the start of offspring development, and represents the key physiological event that induces re-organization and specialization of breast tissue. Such drastic tissue remodeling has also been linked to epithelial cell transformation and the establishment of breast cancer (BC). While patient outcomes for BC overall continue to improve across subtypes, prognosis remains dismal for patients with gestational breast cancer (GBC) and post-partum breast cancer (PPBC), as pregnancy and lactation pose additional complications and barriers to several gold standard clinical approaches. Moreover, delayed diagnosis and treatment, coupled with the aggressive time-scale in which GBC metastasizes, inevitably contributes to the higher incidence of disease recurrence and patient mortality. Therefore, there is an urgent and evident need to better understand the factors contributing to the establishment and spreading of BC during pregnancy. In this review, we provide a literature-based overview of the diagnostics and treatments available to patients with BC more broadly, and highlight the treatment deficit patients face due to gestational status. Further, we review the current understanding of the molecular and cellular mechanisms driving GBC, and discuss recent advances in model systems that may support the identification of targetable approaches to block BC development and dissemination during pregnancy. Our goal is to provide an updated perspective on GBC, and to inform critical areas needing further exploration to improve disease outcome.

## Introduction

Despite significant advances to detect and treat breast cancer (BC), deficits still exist for a subset of cancers arising during or shortly after pregnancy. BC associated with pregnancy—which encompasses tumors that develop during gestation (GBC), lactation, and the post-partum period (PPBC)—remains an elusive disease with incredibly poor detection and prognosis due to a variety of clinical and experimental barriers. While the incidence of BC which develops during or after pregnancy varies between 1:1000 and 1:3000 pregnancies, the exact incidence will likely shift as the definition of PPBC evolves from tumors diagnosed within one year of pregnancy to those diagnosed up to 5–10 years post-partum [1–4]. Such cancer subtypes are more prevalent in younger women compared to BC in general, and it is hypothesized that the drastic changes occurring in the mammary gland during pregnancy, lactation, and involution may be key drivers of neoplastic establishment and/or the progression of pre-existing disease [5–10]. However, it has also been established that pregnancy earlier in life bestows life-long protection against BC [11–13], an effect that collectively controls changes to mammary epithelial cells (MECs), and alterations of mammary gland immune composition post-pregnancy [14–16]. Together, these observations suggest the window of opportunity for the establishment of cancer may be more limited to the period around and shortly after pregnancy [15, 17–19]. Consistent with this notion, it has been shown pregnancy elicits an increased risk of the development of BC in the short-term, and thus warrants further study into how gestational and post-partum alterations influence BC [6, 20, 21]. Therefore, to better understand the drivers of GBC and PPBC, it is critical to identify stromal changes to breast tissue which may contribute to neoplastic development.

## Characteristics and Composition of GBC and PPBC 

### Incidence and Prognosis

According to the National Cancer Institute, BC is the most common cancer diagnosed during pregnancy (about 1 in 3,000 pregnancies), accounting for up to 2–4% of cancer occurrence [22–24]. While BC incidence during pregnancy or the post-partum period is uncommon, patient diagnosis has increased over decades and is anticipated to continue increasing as women delay having children. One retrospective study compared women in China diagnosed with BC during or up to 1 year after parturition with age-matched, non-pregnant patients and showed patients with pregnancy-associated cancer had a shorter cancer-free survival period (32 months versus 37.5), a doubled rate of recurrence (25.4% versus 12.7%), and a more than doubled death rate (20.6% versus 7.9%) compared to the age-matched group [25]. Another study conducted in Saudi Arabia found similarly reduced survival rates associated with pregnancy, with a 5-year survival rate of 82% in non-pregnant patients compared to 65% in pregnant patients [26]. While these data vary by region and cohort size, several other studies have linked pregnancy to worse outcomes in BC and warrant further attention specifically to GBC [27–32]. In fact, one study found BC to be one of the only pregnancy-associated cancers in which lactation specifically doubled the cause-specific death risk (Hazard Ratio of 1.95) [33]. Yet, the reasons behind the poorer prognosis are debated; many groups suggest late diagnosis is a main factor behind the differential outcome [1, 34, 35]. Moreover, it is likely the stage of pregnancy at disease inception influences progression, yet little has been done to stratify BC based on pregnancy status, or to profile the differences between cancers arising during the gestation, lactation, or involution phases of pregnancy.

### Clinical Presentation and Detection

One of the longest standing methods for BC detection is palpation. But in light of the vast changes occurring within breast tissue during pregnancy—as well as the general younger age of patients during pregnancy—it is of little surprise that many typical signs of BC may go unnoticed, or be attributed to side effects of breast maturation and milk letdown [4, 36–38]. Moreover, according to the CDC and other sources, many symptoms of pregnancy are similar to the warning signs of BC (Table 1) [36, 37, 39–41]. Adverse effects from milk production and breast feeding, such as clogged milk ducts or mastitis, may further exacerbate breast pain, discharge, and nodularity, and could delay BC diagnosis [1, 4, 34, 36, 38, 40–44]. Interestingly, one study which interviewed patients with BC determined 90% of patients diagnosed during pregnancy or up to one year post-partum presented with a palpable mass, yet 60% of these patients experienced a delay in diagnosis compared to 11% in cases which did not involve pregnancy or lactation [34]. The primary reason for delay reported in pregnant and post-partum patients was attributed to physician’s reassurance, which was not reported at all as a reason for delay in cases which did not involve a current or recent pregnancy (40% versus 0%) [34].

**Table 1:** Symptoms common to both breast maturation during lactation and the development of breast cancer. (A) A list of commonly reported symptoms associated with pregnancy and breast feeding. (B) Similar symptoms used as warning signs to diagnose breast cancer

A. Pregnancy and Breast Feeding	B. Breast Cancer
Full, stiff, warm feeling [40]	Thickening or swelling of the breast [41]
Engorgement [40]	Size and shape changes [41]
More nodular, dense tissue [36, 37]	New lump in breast [41]
Thick, yellow colostrum secretion [40]	Nipple discharge [41]
Bloody nipple discharge [36]	
Milk leakage [40]	
Sore nipples, discomfort [40]	Pain in nipple area [41]
Pain while breastfeeding [40]	Pain in breast area [41]
Flaking or cracking of skin on nipple [40]	Irritation of skin [41]
Darkening of nipples, areolas, veins [39]	Redness or flaky skin [41]

While detection modalities are presently available for diagnosing generalized BC, and their use has improved patient outcome, several of these are not available to women during gestation due to potential adverse effects on fetal health. For example, non-pregnant patients are eligible to receive both contrast and non-contrast magnetic resonance imaging (MRI) as a way to detect both primary and metastatic lesions across various tissues, including bone. However, contrast MRI relies on radioactive gadolinium to delineate tissues and identify suspicious regions. As gadolinium is known to cross placental tissues, and therefore has potential adverse effects on fetal health, patients with GBC are ineligible for contrast MRI [36, 37, 43]. Additionally, while patients during lactation are eligible for contrast MRI, they will not be able to breast feed during this window due to transmission of radioactive material via breast milk, which may serve as a barrier to some patients in considering when (or whether) to proceed with MRI. X-rays are also utilized for the detection of tumors and metastases; however, gamma radiation can cause permanent damage during fetal development, and thus should only be used in combination with lead shielding of the abdominal cavity [24, 43]. In this way, lesions located in the lower abdomen may go undetected. A more localized way of detection used commonly to screen and monitor women for BC later in life (ages 50–74) is mammography. Generally, mammograms are not performed in younger patients, and are further not recommended during pregnancy unless other methods of detection have already been employed to confirm suspicion [24, 36, 37]. Additionally, studies have shown mammogram reliability is decreased during pregnancy and lactation due to increased breast density and the accumulation of milk in breast tissue, with sensitivity reported as ranging between 78–90% [37, 42, 43].

The gold standard of detection for pregnant patients is ultrasound, which is nearly 100% effective in detecting masses localized to the breast, and is a reliable, non-invasive diagnostic tool [1, 36, 37, 42, 43]. However, the depth with which ultrasounds can penetrate limits the use of sonograms as a method to detect metastases in tissues which are less accessible externally. Moreover, the use of sonograms to screen a broader range of tissues for metastases is not typical. Therefore, it is critical to develop better mechanisms of detection, for example through identification of biomarkers or improved diagnostic tools safe for implementation during pregnancy, as well as through characterization of risk factors for proper assessment. The development of concrete plans to monitor at-risk women throughout and after pregnancy will be paramount in improving outcome for patients with GBC or PPBC.

One study from 2011 which compiled multiple case studies more thoroughly characterized the clinical presentation of BC during or shortly after pregnancy. Of 481 cases examined, only 8% of patients presented with pain, 4% with swelling, and < 3% with bloody discharge, suggesting clinical assessment based on the canonical symptoms of BC is insufficient to discern between pregnancy-associated breast changes and cancer [1]. Indeed, delay in diagnosis due to inaction and failure to perform additional screening and profiling has been documented for decades, yet the way pregnant and post-partum women are assessed and cared for has not changed [1, 34, 35]. It is evident we must re-evaluate the treatment of pregnant women, particularly those presenting with persistent lumps, regardless of the existence of secondary symptoms. Given ultrasound assessment of lumps is essentially 100% effective in identifying suspicious masses, it is unclear why ultrasound followed by ultrasound-guided biopsy when warranted have not become a routine assessment during early pregnancy or post-partum lactation and involution, particularly amongst higher risk individuals, like women with a familial history of BC, or those carrying BRCA mutations, who are or were recently pregnant.

### Subtype

GBC and PPBC cases are represented by all clinical subtypes of BC, with about ~ 50–60% of cases classified as triple negative disease (TNBC) [45–50]. This statistic is concerning given the therapeutic barrier triple negative status poses, as well as the high rate of TNBC relapse [51, 52]. According to the American Cancer Society, TNBC has a 5-year survival rate of 91% for localized disease, 65% for regionally spread tumors, and 12% for TNBC with distal metastases [53]. Therefore, further investigation into whether triple negative status during pregnancy is a common feature of GBC is warranted. Methods to profile the molecular characteristics of GBC often rely on determining estrogen and progesterone receptor status. Yet, given the increase in estrogen and progesterone availability during pregnancy, it is possible the triple negative status associated with pregnancy is simply an artifact of receptor saturation and/or subsequent downregulation during pregnancy [1, 54]. Deeper genetic and proteomic profiling of GBC is thus needed to definitively show gestational cancers are often hormone receptor negative, confirming a proclivity of GBC toward the triple negative phenotype. Importantly, as treatment scheme is often contingent on receptor status in BC, this distinction and assessment will be crucial to making informed plans for the treatment of GBC. The clinical ramifications of GBC assuming a triple negative phenotype are further discussed in Sect. 3.7.

### Biophysical Environment

Of particular relevance to BC is stromal extracellular matrix (ECM). A structural component of tissues, ECMs like collagen, laminin, fibrin, and hyaluronic acid are found in relatively low levels in soft, normal tissues. ECMs are organized into distinct patterns which serve to direct cell behavior, phenotype, polarity, and organization [55–59]. For example, mouse studies have shown that the remodeling of ECMs is essential for ductal morphogenesis in nulliparous mice and during early pregnancy [56, 60–64]. In fact, mice deficient for remodeling enzyme TIMP1 display enlarged TEBs, suggesting there are spatial cues ECM provides to physically guide the size and shape of mammary ducts [60]. Indeed, physical restriction via ECM has been posited as a potential mechanism to prevent convergence of ductal structures within the developing mammary gland, and ductal branches have been observed to bifurcate around obstacles [56, 65]. Other studies have confirmed ECM distribution and physical arrangement influences ductal branching and elongation both ex vivo and in vivo, and that targeting pathways involved with the transduction of these external cues (i.e., integrins, Rac, Rho, ROCK) abrogates proper organization and development of terminal end buds [55, 56, 59, 66–69]. Further, ECM has been implicated in the maintenance of alveolar development, differentiation, and function during pregnancy.

However, disruption of the extracellular matrix, excessive deposition and remodeling, or the generation of tension on and/or alignment of fibrous ECMs, plays a role in cancer progression and dissemination [55, 57, 58, 70–72]. Importantly, ECMs are recognized as prognostic factors in BC, as increased and/or aligned collagen is correlated with poor patient prognosis and decreased patient survival, for example [71]. In fact, some studies have shown that risk of BC in women may correlate more strongly with breast density and stiffness than to epithelial density, suggesting stromal ECMs are just as important to cancer progression as the cancer cells themselves [70, 73–75]. Yet ECM composition in the mammary gland across development, lactation and involution, and how the composition, architecture, and remodeling influences GBC and PPBC, has not been well characterized. Schedin and colleagues have conducted foundational research suggesting there are inherent differences in matrices derived from rat mammary glands across various stages of pregnancy [7, 8, 10, 76–79].

Astonishingly, ECM derived from involuting glands increased breast tumor cell migration compared to matrices from any other stage of pregnancy, suggesting physical cues present in involuting glands alone are sufficient to enhance metastatic potential [7, 8, 77]. While studies have not characterized the architecture of these 3D matrices, several groups have shown ECM structure plays a fundamental role in driving directed cell migration by providing contact guidance cues for cells to respond to and migrate along. Interestingly, tumors formed in mice following pregnancy were comprised of up to 44% stroma compared to 9% in nulliparous mice, with an increase in bone marrow-derived myofibroblasts and endothelial cells, higher collagen/ECM content, and significantly elevated angiogenesis [80]. Unfortunately, this study was conducted in NOD/SCID mice, and thus the contribution of an intact immune system to tumor burden and spreading could not be assessed. Nevertheless, this study establishes a clear connection between systemic estrogen signaling during pregnancy, tumor progression, and stromal dysplasia. It is possible ECM architectures present in involuting mammary glands are distinct from those in other stages of pregnancy and lactation, and serve as a conduit to facilitate epithelial dissemination. This could hint as to why cancers discovered during the post-partum period appear phenotypically different than those diagnosed during pregnancy. Given the alterations to ECM composition and architecture known to occur during pregnancy and involution, the connection between ECM and cell migration, and the similarities between parity-induced and cancer-associated microenvironmental changes, it is likely ECM informs GBC progression and metastasis.

### Metastasis and Recurrence

GBC and PPBC spread to the same canonical sites as BC more broadly, suggesting the inherent nature of disseminating cancer cells to home to particular organs is conserved despite parity. However, GBC and PPBC tend to metastasize earlier and at a higher frequency, with majority of cases presenting as late stage invasive carcinoma with lymph node involvement [3, 5, 81]. It is likely the delayed detection of BC during pregnancy, as well as GBC’s tendency to be triple negative, both contribute to enhanced metastasis. Further, given EMT and motility profiles inherently increase during mammary gland maturation, and the implications EMT has in establishing metastases, it will be crucial to characterize the EMT state of MECs across gestational stage [82–87]. Three-dimensional organoid models have attributed murine TEB invasion to collective cell migration, and have revealed that cells within TEB caps display a more mesenchymal, migratory phenotype consistent with EMT [85–87]. EMT is a well-established switching of cells from an anchored, polarized state toward a more migratory state, and plays a crucial role in the organization of normal tissues as well as the spreading of cancer cells [82, 83, 86]. In non-neoplastic mammary epithelial cells specifically, EMT promotes more stem-like characteristics, and genes associated with EMT are increased in cells within TEBs [85, 87–89]. Therefore, an understanding of how normal breast maturation during pregnancy influences EMT and cell motility may lend to our understanding of how pregnancy primes epithelial cells for pre-malignant behavior prior to transformation. It is possible the attenuation of EMT and epithelial motility in high risk patients, or those with existing disease, could improve prognosis for individuals with GBC or PPBC. Moreover, it is likely EMT changes as a response to hormone signaling, yet whether hormonal fluctuations during pregnancy, lactation, and involution truly influence metastasis is unknown. Along these lines, it is completely unknown whether systemic pregnancy signals prime metastatic niches to support tumor cell seeding and growth. GBC and PPBC are known for possessing a higher likelihood of disease relapse later in life [3, 6, 27, 81, 90]. It has been posited that, due to the younger age range of individuals diagnosed with GBC and PPBC compared to BC more broadly, there is an increased risk for disease recurrence; whether there are other lasting alterations which influence risk of recurrence is unclear. Given that metastasis and disease relapse are the two main drivers of cancer related deaths, it will be imperative to understand how parity influences these processes.

### Therapeutic Availability

While strides have been made to improve treatment options and efficacy for patients with BC, there are still prominent treatment deficits when it comes to GBC. Most therapeutic options are not available to GBC patients, and the utility and plausibility of these options is dependent on the time of diagnosis, the window before delivery, etc. Below, we provide a comparison of several therapeutic options available to patients with BC, and put these into the perspective of GBC (Table 2). Many of these options are either not recommended, or are accompanied by serious complications and contraindications for patients with GBC [91, 92]. Further, while there are several chemotherapies safe for administration later in gestation, many are contraindicated after 35 weeks of gestation, and up to 3 weeks post-delivery, due in part to a detrimental impact on maternal blood cell count, which influences maternal immunity and increases likelihood of excessive bleeding. Further, there is concern that many of these options may promote premature delivery, and thus are generally not given during the window just preceding birth. In fact, many of these options are limited to the second and third trimester, or strictly during the post-partum period. This is of special concern given that, according to a 2021 Dutch study, nearly 75% of GBC and PPBC cases were diagnosed during pregnancy specifically, rather than during the post-partum period [31]. It is evident that many of the gold standard chemo- and radiation therapies are simply not an option for women who are pregnant, which could also exacerbate the prognosis for GBC. Even during the post-partum period, significant consideration must be dedicated to determining how the course of treatment will impact the patient’s ability to breastfeed, and any potential risks which may come to the infant as a result. Of note, many of our most robust treatments, like radiation, tamoxifen, and trastuzumab, are not recommended during pregnancy. For example, immunotherapy has become an attractive method of tumor targeting and has shown success in treating several tumor types reviewed here [93, 94]. However, the utility of immunotherapy during pregnancy is unclear, thus immunotherapies remain contraindicated [95, 96]. Potential complications are outlined in this review [96], which highlights how perturbing immunity may come with serious consequence to the tolerance established between mother and fetal immunity during pregnancy, along with other reported and speculated consequences.

**Table 2:** Interventional strategies presently used for generalized BC, and their clinical utility in treating GBC. Below is a compiled list of both physical and chemical treatment regimes available for breast cancer, their mechanism of action, as well as their utility specifically for patients with GBC and the gestational stage (“GS”) at which GBC patients are eligible for this treatment option through trimesters 1–3, or post-partum (“PP”). Potential complications which may arise as a result of the surgical or chemical intervention are also highlighted

Treatment	Mechanism	GBC use	GS	Complications	REF
**Radical mastectomy**	Surgical removal	High	1 +	Miscarriage/abortion	[47, 91, 97–99]
Paclitaxel Docetaxel Vinorelbine	Microtubule disruption	Yes	2 +	Early delivery Hypersensitivity Limited data	[47, 91, 100–104]
Cyclophosphamide	RNA/DNA synthesis: DNA crosslinker	Yes	2 +	Early delivery Myelosuppression	[91, 97, 99, 103–105]
Doxorubicin	RNA/DNA synthesis: Topoisomerase inhibitor	Yes	2 +	Early delivery Cardiac toxicity Tumor lysis syndrome	[91, 97, 99, 104, 105]
Flouracil/5-FU Capecitabine	RNA/DNA synthesis: pyrimidine analogue	Yes	2 +	Early delivery Hand-foot syndrome GI effects	[91, 97, 99, 100, 103, 105, 106]
**Breast Conservation**	Partial removal with reconstruction	Rare	3 +	Incomplete removal Must be followed up with chemo within 6 weeks	[91, 97, 107]
**Radiation**	DNA damage	**No**	PP	Miscarriage/abortion Birth defects Childhood cancer	[47, 97, 98, 108]
**Hormone therapy** • Tamoxifen • Anastrozole • Letrozole • Exemestane • Fulvestrant	ER + and/or PR + tumor cells	**No**	PP	Birth defects Fetal toxicity Limited data	[47, 91, 98, 109]
**Targeted therapy** • Trastuzumab • Pertuzumab • Tucatinib • Neratinib • Lapatinib	Her2 targeting	**No**	PP	Fetal toxicity Limited data	[47, 91, 100, 101]
Immunotherapy	Immune modulation	**No**	PP	Limited data	[95, 96]
Methotrexate	RNA/DNA synthesis: Nucleotide synthesis	**No**	PP	Teratoma formation Miscarriage/abortion	[47, 91, 97, 104]
Carboplatin	RNA/DNA synthesis: DNA crosslinker	**No**	PP	Limited data	[104, 110]
Epirubicin	RNA/DNA synthesis: DNA intercalation	**No**	PP	Limited data	[97, 100, 104, 111]
Everolimus	mTOR inhibitor	**No**	PP	Limited data	–
Palbociclib	CDK4/6 inhibitor	**No**	PP	Limited data	–

Further compounding this lack of sufficient treatment options for GBC is the consideration that triple negative cancers and metastatic disease are both in need of better treatment options as is, regardless of gestational status [112]. Emerging therapies with success in targeting TNBC are not available to patients during pregnancy. For example, PARP inhibitors have shown utility in treating TNBC, but their administration during pregnancy is not recommended for multiple reasons, including lack of data regarding safety during pregnancy, speculated fetal toxicity, and uncertainty of transmission across breast milk [113, 114]. However, studies in mice have shown that inhibition of PARP in vivo results in loss of pregnancy, ovarian toxicity, and may have potential impacts on fertility [115, 116]. Ultimately, we speculate the propensity of GBCs to have already spread, and to assume a triple negative phenotype, worsens prognosis.

Even therapies which are amenable to pregnancy may still be accompanied by severe side effects to both maternal and fetal/infant health. Given the uncommon incidence of GBC and PPBC, we cannot rely solely on data gathered for GBC and PPBC as many studies involve a case study of one or few individuals. Rather, the field should look more broadly at cancers diagnosed during pregnancy, and the adverse effects arising as a result of various treatment schemes. However, one such study regarding the safety of immunotherapy during pregnancy raises a critical point that pregnant women or women who become pregnant during clinical trials are often excluded and/or removed from participating due to concerns or uncertainty of fetal harm, thus limiting our understanding of how many emerging therapies may impact fetal and infant health [95]. In this same vein, another study of cancers diagnosed during pregnancy, which contained follow up with patients and infants from over 16 countries, found connections between therapies and adverse outcomes directly related to maternal and fetal health [117]. For example, they determined a correlation between platinum-based chemotherapies and a small infant size for gestational age, as well as a correlation between taxane administration and infant NICU admission [117]. Further studies may serve to link specific treatment regimens to likely clinical adverse reactions, and inform strategies for follow up with and care for patients on these regimes. Regardless of therapy, however, the most common issue across cancers during pregnancy was preterm delivery; in BC specifically, majority of live births occur prematurely, with only 37% falling at or after 37 weeks. Thus, it is paramount to continue to examine both maternal and fetal health as we build more robust treatment schemes. As many therapies have utility across various cancer contexts, further literature reviews to assess treatment schemes and success during pregnancy may help to inform new strategies to treat GBC.

## Re-Evaluating the Post-Partum Window for PPBC

The traditional definition of BCs associated with pregnancy encompassed tumors identified during pregnancy, or within the first few years after delivery. However, multiple studies point to the post-partum period stretching as long as 5–10 years depending on maternal age at delivery [3, 5, 81], with parous patients exhibiting gene signatures distinct from nulliparous women [118]. In fact, gene analysis has revealed an increased tendency toward high grade TNBC for PPBC, a signature maintained up to 10 years after pregnancy [5, 47, 48, 118]. Moreover, stromal changes which occur during involution, like the influx of immunosuppressive cells, have been shown to persist after delivery, although the longevity of these changes is unknown [119]. Therefore, we echo here a familiar sentiment that the post-partum period for diagnosis should be re-evaluated in order to better inform treatment schemes for PPBCs, and follow up for high risk patients [2, 3, 9, 20]. Currently, the scope of knowledge regarding PPBC, patient outcome, and treatment efficacy is limited to a very narrow window during which cancer arises. Given there may be cases falling into the 1 to 10 years post-partum period which are more phenotypically similar to PPBC, and less similar to generalized BC, there is likely an underdiagnosis of patients with PPBC who may actually benefit from more rigorous follow up and aggressive treatment schemes than age-matched nulliparous women with BC.

## Potential Models to Study GBC and PPBC

### Cell Lines and Culture Systems

**Fig. 1:**
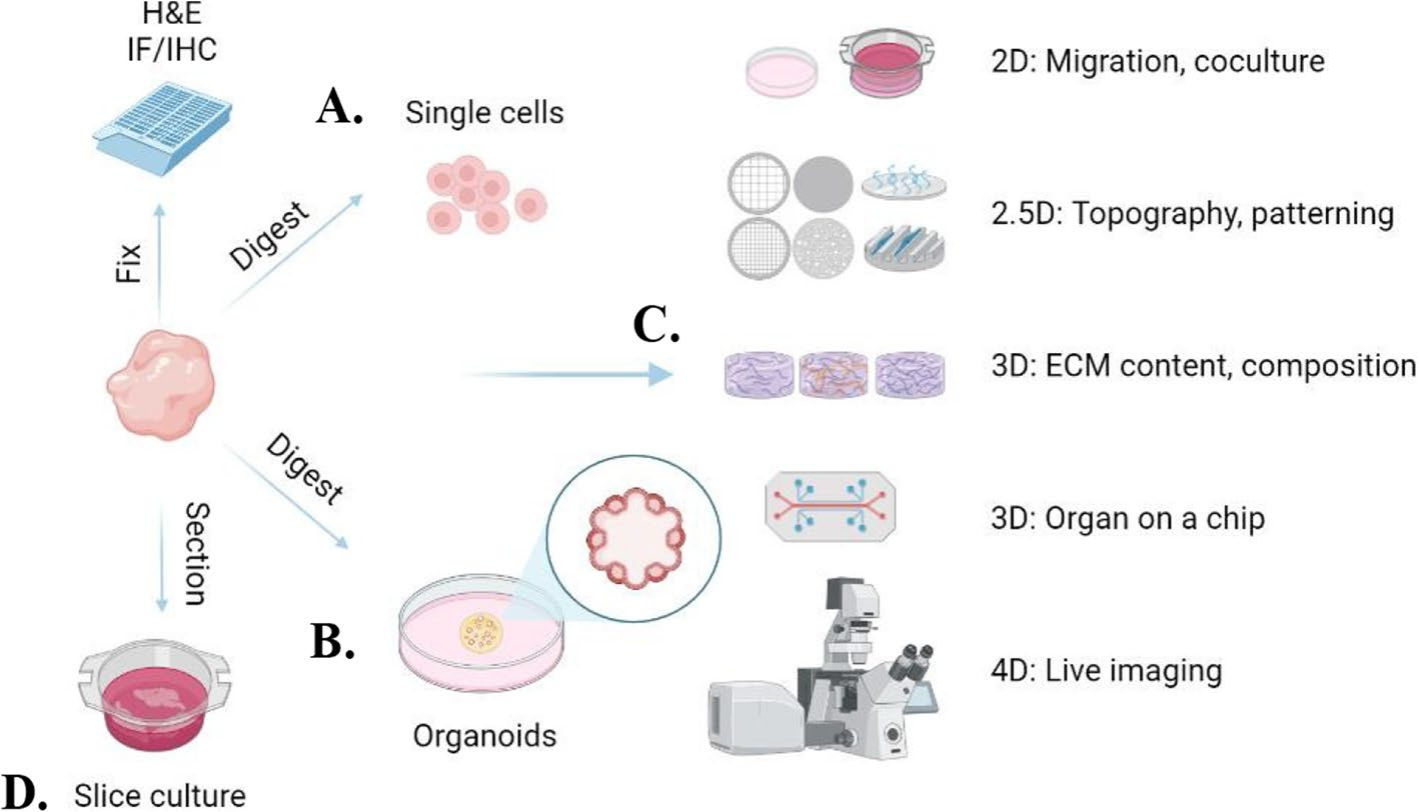
Complex culture systems available to study GBC and PPBC. A schematic of how live tissue samples can be processed, cultured and utilized for subsequent characterization and analysis. Tissues can be isolated and fixed, embedded, and sectioned for staining. For culture, and dependent on protocol and subsequent experiments, tissues can be digested into single cells (**A**) or organoids (**B**), which can then be used in complex culture systems (**C**), varying in complexity from 2D cell culture and co-culture, to live imaging of cells and organoids in 3D environments. Additionally, tissues may be sectioned using a vibro-, cryo-, or microtome for subsequent culture and analysis (**D**)

While common cell culture systems and assays, like 2D cell culture, transwells, and scratch-wound assays, are widely used in modern research, the emergence of 3D primary organoids, coupled with live imaging, single cell sequencing, and other cutting-edge tools, may provide a clearer scope of how cancer cells respond to their surrounding environment (Fig. 1). For example, models of migration which utilize 3D matrices can provide 4D information by using live imaging to quantify the spatiotemporal dynamics of cells in response to, in example, matrix composition. Matrix composition and stiffness can easily be tuned in 3D to determine which matrices facilitate tumor cell extrusion. Multicellular spheroid and organoid systems could also represent ideal strategies for studying GBC and PPBC. For example, 3D organoid models exposed to pregnancy hormones have been shown to recapitulate the epigenetic, molecular, and phenotypic changes which occur during pregnancy [120]. Such models, established from various mouse strains, and from normal and neoplastic mammary tissues, may be valuable for investigating a variety of processes including cellular invasion, heterogeneity, chemoresistance, or interaction with stromal cells and ECMs. Implementation of emerging strategies like these may better recapitulate in vivo features of mammary gland tissue for the study of GBC and PPBC progression.

Additional models, like “tumor on a chip,” can be used to mimic multiple points of the metastatic cascade [121–124]. For example, one study established a model of chemical crosstalk between three compartments known to play a role in BC metastasis. By culturing tumor, neural, and bone compartments in chemical communication, this study revealed novel insight into how chemical communication influences tumor progression [125]. However, this system does not facilitate physical interactions between tumor cells and other compartments like the vasculature. By using shear fluid force to simulate circulation, channels on chips can be “vascularized” by flowing through endothelial cells. This variation of microfluidics could be used on a basic level to generate vascular permeability mimicking that during a pregnant versus non-pregnant state to study tumor cell intravasation and extravasation as a function of pregnancy hormones. Systems could be further elevated by adding niche-specific cell types and/or ECMs to study specific tumor cell seeding in metastatic sites from GBC and PPBC. Indeed, work from the Kamm lab has explored biomimetic microenvironments recapitulating bone and muscle to understand metastasis [126]. Systems like this would facilitate a deeper understanding of tumor cell tropism and preferential homing to metastatic sites in response to pregnancy, and may hold utility in predicting individual patient tumor behavior and interventional success. In line with this, the Konstantopoulos group established a device which could be applied to predict patient-specific metastatic potential and response to antimetastatic therapeutics [127]. This could be applied to assess risk and patient response ex vivo, and may be encouraging for pregnant patients in their decision to move forward with treatment at earliest eligibility. The tunability of this system would essentially allow for study of multiple stages of pregnancy, with multiple cell types simultaneously.

Even traditional 2D cell culture has been elevated to allow study of how biophysical aspects of GBC and PPBC may impact progression. For example, Engler et. al. showed in 2006 that substrate stiffness can regulate the fate of mesenchymal stem cells, with matrices mimicking bone, muscle, and brain being osteogenic, myogenic, and neurogenic, respectively [128]. Yet, despite the clear role mechanics play in directing cell behavior and phenotype, little work has been done to understand how stiffness, or mechanical changes during pregnancy like elevated fluid pressures and increased breast density, would impact GBC and PPBC progression and invasiveness, or preferential homing during metastasis, for example. Additionally, the use of polyacrylamide 2D substrates would allow study of how stiffnesses reminiscent of canonical BC metastatic sites influence tumor cell seeding with and without pregnancy hormones. Along these lines, mechanical cues like matrix alignment have been shown to be important to BC progression, metastasis, and recurrence, but have been largely unexplored in the context of pregnancy [57, 71, 129]. The utilization of “2.5D” nanopatterned flat substrates would allow for perturbation of whether contact guidance cues alone or in addition to pregnancy hormone signaling encourages epithelial cell directed migration and elongation. More complex systems which couple nanotopography with other biophysical cues like substrate elasticity may provide a deeper understanding of the individual and additive impacts of ECM composition and matrix mechanics on GBC and PPBC progression, particularly from the lens of pregnancy-induced breast stiffening. For example, systems like those utilized by Tabdonov which combine matrix stiffness, alignment, and ECM composition could reveal how the patterning of specific ECMs in distinct topographies influences BC cell migration in the context of pregnancy [130].

### In vivo Models

**Fig. 2:**
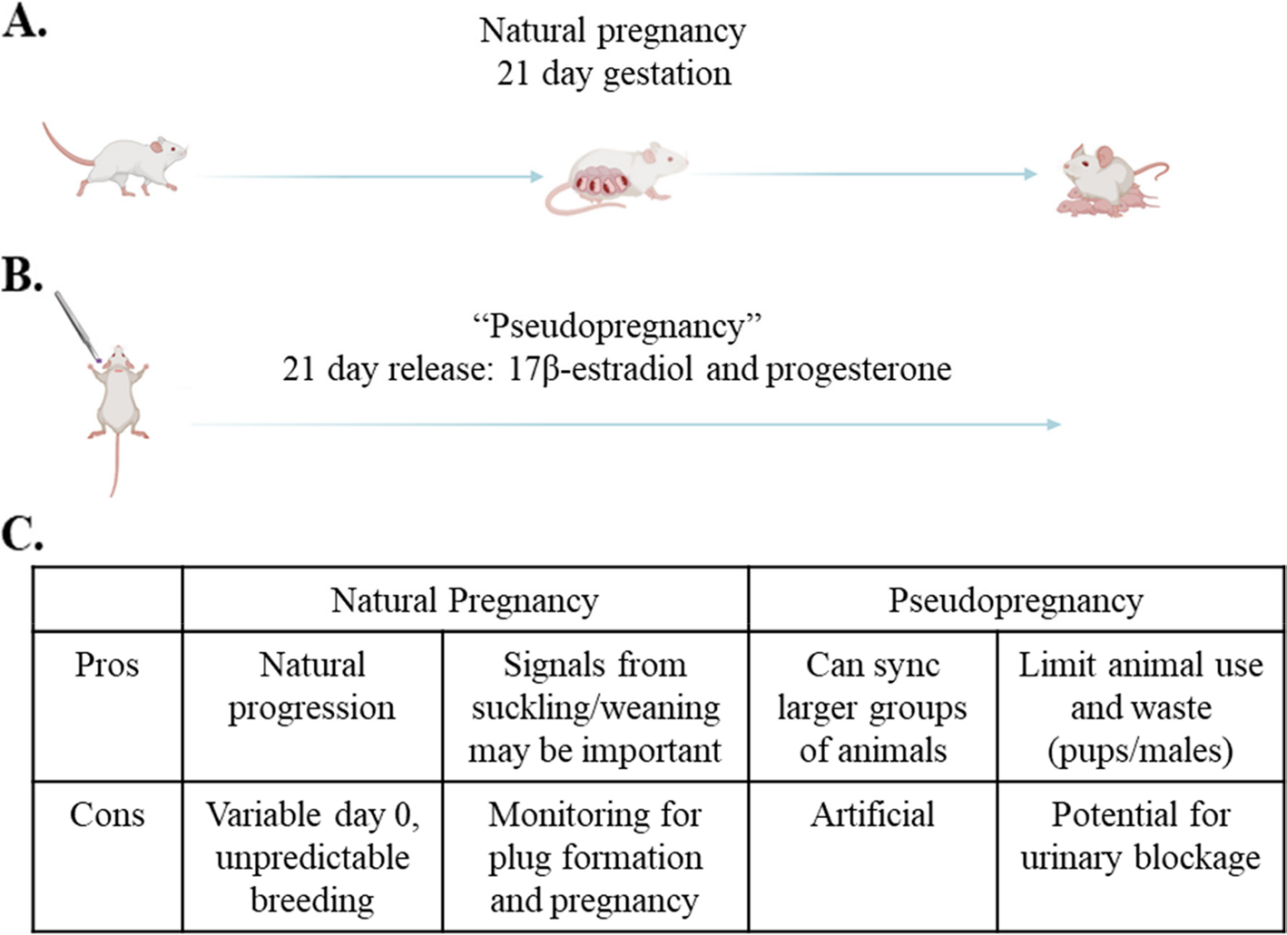
Using mice to study the impact of gestation and pregnancy-associated hormones in vivo on breast cancer. **A** Natural pregnancy can be employed to study how pregnancy, and the various stages of gestation, influence mammary gland development, function, and reversion during pregnancy and the post-partum period. **B** Surgical implantation of a 21-day slow release estrogen/progesterone pellet can be used to mimic pregnancy via the systemic release of pregnancy-associated hormones, which drive changes to mammary tissue consistent with natural pregnancy. **C** Potential pros and cons to both models which may dictate which will be better-suited for studies of pregnancy, GBC and PPBC

Given their relatively short gestational cycle, mice provide an excellent model of pregnancy for the study of GBC and PPBC, by allowing the analysis of how cancer cells develop and disseminate during and after pregnancy (Fig. 2A). However, as the exact start of pregnancy can be difficult to pinpoint, researchers could employ models of “pseudo” pregnancy as an alternative to robustly characterize changes to tissues in response to pregnancy hormones (Fig. 2B). The implantation of 21-day slow release pellets containing 17-β estradiol and progesterone, or vehicle control, allows the study of how hormones influence tissue composition in a more tunable, controlled manner, while mimicking the molecular and cellular alterations during pregnancy-induced mammary development [131]. While findings from pseudopregnancy models should ultimately be validated using pregnant animals, hormone systems provide a reductionist approach to understanding the contribution of estrogen and progesterone to GBC progression. Both true pregnancy and pseudopregnancy have potential benefits and pitfalls (outlined in Fig. 2C), but can be used across a wide range of animal strains, including spontaneous or inducible models of mammary oncogenesis, or in pregnant or post-pregnancy animals injected with tumor cells.

Many preclinical mouse models exist for the study of BC, and many involve the dysregulation or deletion of pathways canonically seen in clinical BC [132–134]. Earlier models employing immunocompromised mice, or performing subcutaneous tumor cell injections, have provided a solid foundation for in vivo experiments. However, as research has revealed the critical role of the immune system in cancer development, as well as the contribution of organ-specific tissue resident cells, the development of genetically engineered mouse models (GEMMs) have allowed for more translational study of tumor development and pathology. Importantly, GEMMs allow for study of dysplastic tissues which have not fully progressed into neoplasia, giving us a better understanding of early disease establishment.

The most classical BC-GEMMs were developed via the manipulation of cancer-relevant gene function under the control of mammary-specific promoters like the mouse mammary tumor virus long terminal repeat (MMTV-LTR), Whey Acidic Protein (WAP) and b-Lactoglobulin (BLG) promoters, which largely give rise to mammary-specific tumors with histopathological features replicative of human disease [133–137]. Interestingly, the activity of these promoters is enhanced by steroid hormones present during pregnancy and lactation, thus potentially allowing the investigation of how pregnancy-induced changes intersect with those inducing tumorigeneses, and represent an opportunity to study GBCs developing during pregnancy (MMTV), or PPBCs after lactation (WAP, BLG) [138]. To date, models of mammary tumorigenesis initiation and development have been developed to manipulate the function of cancer-relevant genes, including those based on inducible oncogene gain-of-function, or cancer suppressor loss-of-function [16, 132, 139, 140]. Given their already characterized tumor subtypes in non-pregnant mice, utilization of such models to elucidate the basis of cancer development during pregnancy, lactation, and involution, via natural breeding or the use of slow-release hormone pellets, may represent a suitable platform to define intrinsic characteristics of GBC and PPBC.

However, all of these models pose additional obstacles to the study of GBC and PPBC due to the effect that pregnancy and lactation have on augmenting MMTV, WAP and BLG activity [138]. Use of models relying on these promoters therefore diminishes the opportunity to tease apart epithelial dynamics differentially regulated across parity-associated development, and those associated with cancer. To circumvent this, many groups have employed an array of different transgenic approaches to study the effects of pregnancy on mammary oncogenesis. Feigman et al. employed a model of cMYC overexpression under the control of the synthetic cytomegalovirus immediate enhancer/β-actin (CAG) promoter, a doxycycline-inducible model which is not dependent on pregnancy and lactation [139]. Additional models include those implementing localized viral delivery of oncogenes to mammary gland epithelia through intraductal injection. As an example of the utility of this approach in GBC, Haricharan et al. revealed a relationship between pregnancy hormones, epithelial STAT5 signaling, and tumor progression [141]. This study shows pregnancy exacerbates BC risk and progression after viral delivery of oncogenic Erbb2 and Wnt1 to murine mammary epithelia in an MMTV-*tva* mouse model [141]. It is likely models like these, whose activity is not inherently perturbed by gestational hormone signaling, would be beneficial in unmasking pregnancy-related changes to epithelial cell behavior.

**Fig. 3:**
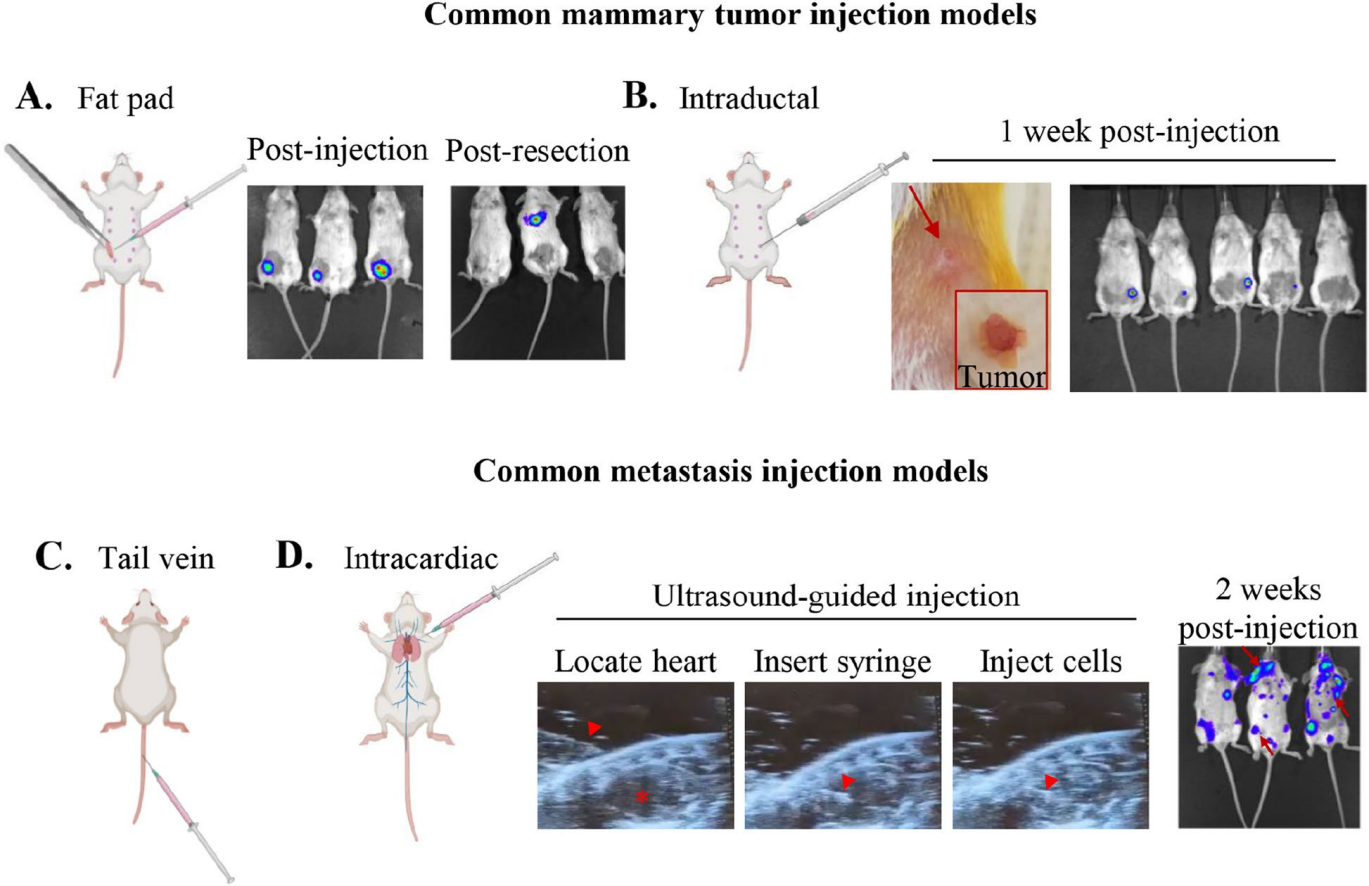
In vivo injection models to study breast cancer and metastasis. A summary of a few commonly used injection models for studying primary mammary tumors (**A**, **B**), and metastatic seeding and colonization from cells in circulation (**C**, **D**). **A** Injection of tumor cells directly into the mammary fat pad can be used to study primary tumor growth and spread from the mammary gland. Luciferase can be a useful mechanism to monitor cell spreading, or success of resection procedures. Requires opening of the abdomen and exposure of the gland to the outside. **B** Intraductal injection, or delivery through the nipple and mammary ductal tree, can provide a less traumatic alternative as it does not require surgical opening of the animal to deliver tumor cells to the mammary gland. **C** Tail vein injection of tumor cells into circulation is a commonly used model of metastasis, often resulting in lung metastases. **D** Ultrasound-guided intracardiac injections involve using an ultrasound probe to locate the left ventricle (asterisk), guide a syringe (arrow head) to the heart, and inject tumor cells into circulation. IC injection results in whole-body metastatic seeding to clinically relevant sites including the bone, lung, and brain (canonical breast cancer metastatic sites, arrows)

In addition to GEMMS, fat pad injections, or the delivery of tumor cells directly to the mammary gland, can provide a good model for tumor development (Fig. 3A). In fact, such approach has been utilized to understand how post-partum alterations to the mammary gland support the development of tumors using well-established murine cell lines [142]. However, this method requires surgical procedures which could trigger inflammatory responses, perhaps altering the progression of tumor development. As a counter approach, the delivery of cancer cells via intraductal injections could represent a suitable system for the study of mammary tumor development (Fig. 3B). In this way, tumor cells can be delivered directly to the gland in a more controlled fashion, and likely allow tumors to form more uniformly in terms of physical location. Regardless of the preferred route of delivery, the injection of mammary cancer cell lines and/or organoids derived from GEMMs into the tissue of mice during pregnancy, lactation and involution may facilitate an understanding of tumor cell-induced alterations to gland microenvironment, and their importance in GBC and PPBC progression.

Importantly, tumors which form after injection could be resected at various time points during pregnancy or the post-partum period as a way to better mimic what would happen clinically in patients with GBC or PPBC. By mimicking this resection in mice, one could study the dynamics of disease recurrence, metastasis, and dormancy. Additional methods to study metastasis directly include tail vein injection and ultrasound-guided intracardiac injections (Fig. 3C and D). The former largely results in lung metastases, a hypothesized artifact of cells becoming physically lodged in the small capillaries of the lung. Thus, it is unclear whether tail vein delivery of tumor cells facilitates preferential homing of cells to the lungs, or rather results in the unavoidable obstruction of tumor cells and their subsequent colonization in the lung. Intracardiac injections have been brought to light as a potential alternative, giving rise to whole-body metastases consistent with preferential homing of the tumor type based on clinical trends. However, intracardiac injections are incredibly difficult to reproduce and confirm without the addition of ultrasound guidance. By utilizing an ultrasound probe, one can easily and reproducibly locate the heart, bring the syringe with tumor cells to the heart, and confirm the success of the injections (Fig. 3D). Importantly, all of the mentioned injection models can be paired with cell lines tagged with fluorescent or luminescent reporters to allow for non-invasive monitoring of tumor growth and spreading over time (Fig. 3A-D). In example, D2A1s expressing luciferase were injected into virgin Balb/C mice and were seen to metastasize to the bone, brain, thymus, lungs, and liver upon intraperitoneal injection of substrate D-luciferin (Fig. 3D). These metastatic sites are consistent with clinical trends seen in BC patients and, thus likely more translationally relevant compared to tail vein injections. We therefore propose intracardiac models be used instead of, or at least in parallel to, tail vein injection to study metastatic colonization in future BC, GBC, and PPBC studies.

## Discussion: Where do we go from here?

It is unquestionable the urgent need for clinical improvements in the risk assessment, diagnosis, and treatment of women with GBC. While some factors clearly exacerbate risk, like carrying BRCA1/2 mutations, or age at first pregnancy, there inevitably are other risk factors we are unaware of thus far which could help inform follow up for pregnant and nursing women who are likely to develop GBC or PPBC. Breast ultrasounds, while not routinely scheduled for screening pregnant women, are recommended to patients with BC family history or known genetic predisposition to BC. Encouraging high-risk pregnant patients to undergo breast ultrasound may enhance the early detection of breast lesions arising during gestation, thus improving clinical management of GBC. Additionally, the exploration of medications like anti-inflammatories during the post-partum period could serve to help limit immunosuppressive influx and fibrosis during mammary gland involution, which is suspected to be a key driver of PPBC.

Perhaps another more pressing point of discussion for the treatment of GBC and other gestational cancers is the choice between terminating pregnancy to begin aggressive treatment regimens—which may ultimately influence patient survival—or delaying treatment until after delivery, a risk which may endanger both fetal and maternal health. While past studies have suggested the termination of pregnancy does not offer tractable benefit to GBC patients, these studies did not consider possible long-term ramifications of delaying treatment (like disease recurrence later in life). The recent reversal of access to reproductive care in the United States will likely impact patients diagnosed with cancer during pregnancy, as pregnancy ultimately dictates both the timeline and type of therapy administered. Moreover, as some regions of the United States make moves to limit or even ban contraceptives with mechanisms of action which could be considered “abortive,” it is worth questioning how access to chemotherapy during pregnancy may be impacted given several interventional strategies are known to increase risk for spontaneous abortion, miscarriage and pre-term labor. While current treatment strategies to target GBC are largely safe during pregnancy, and do not present great maternal–fetal risk, one cannot rule out the impact of leaving GBC untreated during pregnancy, or of these targeted approaches, on fetal viability. This emotional and physical burden is not alleviated by the current abortion legislation in several countries and states, which prohibit the interruption of pregnancy after 6 weeks of gestation. While there is no link between abortion and BC risk [143, 144], denying cancer care during gestation due to a remote risk of spontaneous abortion would introduce life-threatening risks with harmful—and potentially life-long—consequences to the lives of women. As importantly, denying the option to terminate a non-viable pregnancy in order to begin or continue anti-cancer treatment directly impacts women’s access to treatment procedures to manage GBC. It is therefore paramount to improve 1) the reliability of diagnostic modalities for pregnant patients, 2) the availability of preventative care strategies for high risk patients, including more robust monitoring and follow up during pregnancy and in the post-partum period to promote earlier tumor detection, and 3) the accessibility of safe, efficacious therapies which can be administered as quickly as possible after diagnosis, regardless of gestational stage. Further, given the frequent care throughout gestation and after parturition, it is possible the implementation of additional practices for examining and monitoring the breast tissue may improve early detection and patient outcome.

Overall, there is clearly a lot to improve upon, from basic knowledge of GBC and PPBC risk and establishment, to clinical diagnosis and treatment. It is our hope that this review will highlight caveats and advantages of existing research models so that the field may continue to adapt our approach to interrogating GBC, with the ultimate goal of better understanding the dynamics of gestational cancer, and improving the availability, safety, and efficacy of treatments for women diagnosed with BC during pregnancy.

## Data Availability

Not applicable.
